# Fetal Growth Velocity—A Breakthrough in Intrauterine Growth Assessment?

**DOI:** 10.3390/jcm13133842

**Published:** 2024-06-29

**Authors:** Anna Orzeł, Agnieszka Aleksandra Strojny, Dagmara Filipecka-Tyczka, Arkadiusz Baran, Katarzyna Muzyka-Placzynska, Ewelina Mabiala, Justyna Pajutrek-Dudek, Anna Scholz

**Affiliations:** 1 1st Department of Obstetrics and Gynecology, Centre of Postgraduate Medical Education, 02-097 Warsaw, Poland; anna.k.orzel@gmail.com (A.O.); agnieszkastrojny@icloud.com (A.A.S.); abaran@cmkp.edu.pl (A.B.); zumyka@o2.pl (K.M.-P.); ewelinamabiala@gmail.com (E.M.); j.pajurek@wp.pl (J.P.-D.); 2 Department of Obstetrics and Gynecology, St. Sophia Hospital, 01-004 Warsaw, Poland; d.filipecka@gmail.com

**Keywords:** growth velocity, fetal growth restriction, adverse perinatal outcomes

## Abstract

The pursuit of assessing fetal well-being in obstetrical practice remains a central tenet, propelling ongoing endeavors to explore innovative markers and diagnostic methodologies aimed at prognosing potential perinatal adversities. Deviations from standard patterns of intrauterine growth, whether exhibiting excessive or insufficient trajectories, stand as pivotal indices hinting at underlying pathophysiological processes or heightened concurrent medical conditions. Initiatives like the Delphi consensus and the INTERGROWTH-21st project strive to refine diagnostic criteria and establish international standards for fetal growth assessment. This article aims to present the current knowledge regarding the assessment of abnormal growth, including novel methods such as growth velocity. Integrating fetal growth velocity assessment into perinatal care protocols holds promise in enhancing diagnostic precision. Growth velocity, involving changes in fetal size over a given period, offers insights into distinguishing between constitutional and pathological growth abnormalities. Various methodologies and models have been proposed to evaluate growth velocity, with notable advancements in understanding fetal growth patterns across different trimesters. It is believed that accelerated and reduced growth velocity may be a sensible parameter in the detection of fetal growth restriction (FGR), small-for-gestational-age (SGA) fetuses, large-for-gestational-age (LGA) fetuses and macrosomic fetuses as well as appropriate-for-gestational age (AGA) fetuses that encounter problems with growth continuation. Recent studies found that changes in growth velocity reflect the risk of adverse perinatal outcomes (APOs). Future directions in fetal health research aim to elucidate the long-term consequences of abnormal fetal growth velocity on neurodevelopmental outcomes, highlighting the critical role of early assessment and intervention.

## 1. Introduction

The assessment of fetal well-being remains a substantial goal in obstetrical practice. Current research investigates novel markers and diagnostic tools to predict potential perinatal complications. Abnormal intrauterine growth may indicate ongoing pathologies or enhanced comorbidities. It is considered that the range of normal estimated fetal weight (EFW) lies between the 10th and 90th centiles for gestational age (GA). The standard approach in observing fetuses with abnormal growth involves regular examinations with ultrasound scans (USs), including fetal biometry and Doppler blood flows. Constitutionally small but otherwise healthy fetuses with EFWs between the 3rd and 10th centiles, with normal fetal and maternal Doppler flows, are defined as small for gestational age (SGA) [1].

SGA fetuses are at increased risk of stillbirth and postnatal complications, including hypoglycemia and hyperbilirubinemia. Yet, a more severe growth abnormality related to small size is known as fetal growth restriction (FGR), a nuanced and multifaceted condition characterized by a fetus’s inability to achieve its genetically predetermined potential size. FGR differs from SGA as the condition refers to a more extensive growth restriction (EFW < 3 percentile) or involves abnormal Doppler flows. FGR remains the primary factor of stillbirth and leads to a wide range of potential complications such as preterm birth, low Apgar score and impaired neurological and cognitive development in the future. Harmful consequences of undiagnosed FGR lead to optimizing definitions for better perinatal screening [2].

The opposite factor predisposing individuals to potential adverse perinatal outcomes (APOs) is fetal overgrowth. According to the extent of EFW, an increased fetal size may be diagnosed as macrosomia or large for gestational age (LGA). Fetal macrosomia involves the fetuses with EFW exceeding, depending on the sources, 4000 g or 4500 g regardless of GA. LGA fetuses are those whose EFW exceeds the 90th centile for a given pregnancy week [3]. Adverse ramifications of fetal overgrowth include increased risk of shoulder dystocia, perineal laceration, lack of progress in labor emergency Cesarean section, low Apgar score (below 5 at 5 min), assisted ventilation for more than 6 h, seizure and other neurological complications, significant birth injury or even neonatal demise [4].

A novel parameter assessing fetal surveillance is growth velocity, which encompasses the change in the fetal size over a given period. Determining fetal growth velocity involves successive ultrasound biometric evaluations, as well as EFW calculation. Estimating EFW using growth trajectories is an accurate method for predicting birthweight and the risk of APOs, especially considering an accelerated or reduced fetal growth velocity. Researchers indicate that the parameter may improve the diagnosis rate of late-onset FGR and distinguish the appropriate-gestation-age (AGA) fetuses with reduced growth velocities from the general population. The studies revealed that these two groups demonstrate antenatal and postnatal indicators of placental insufficiency [2,5]. Moreover, reduced growth velocity correlates with the risk of APOs independently of signs of cerebral blood flow redistribution. Fetuses that demonstrated negative growth velocity switched to catabolic metabolism. Discrepancies between EFW and birthweight (BW) are the results of the calculation algorithm or inaccuracies in biometric measurements. However, they can also stem from divergent growth patterns between ultrasound-estimated fetal and actual birthweights [6].

This review aims to consolidate existing knowledge and underscore the significance of the novel parameter fetal growth velocity’s assessment in perinatal care protocols. This article’s second objective is to demonstrate the potential benefits of fetal growth velocity analysis in improving the prediction and prevention of adverse perinatal outcomes. The research in this field is limited, and the presented review may contribute to a better understanding of the role of growth velocity assessment. The reference analysis was conducted through a comprehensive search of multiple academic databases, including PubMed, Medline, Scopus and the Cochrane Library.

## 2. Fetal Growth Restriction—Different Criteria and One Diagnosis?

The underlying causes of FGR are varied, encompassing placental complications, maternal health issues, fetal genetic anomalies and infectious factors. It carries significant perinatal risks, including heightened morbidity and mortality, and predisposes affected individuals to long-term health issues [7]. In childhood and adulthood, persons who have been exposed to FGR are more likely to suffer from cardiovascular, metabolic, neurological or oncological diseases. Complications due to FGR also include impaired psychological and social development, neurologic dysfunction, cerebral palsy, learning problems (reading and arithmetic), lower intelligence level and attention deficit hyperactivity syndrome (ADHD). Moreover, FGR diagnosis was associated with a higher rate of mental disorders, such as schizophrenia, depression, anxiety and bipolar disorder, in the future [8,9].

Managing FGR demands rigorous fetal monitoring and strategic delivery timing to minimize risks. The medical community, however, grapples with achieving a consensus on defining FGR, attributed to the challenge of differentiating between naturally small but healthy fetuses and those undersized due to pathology. Moreover, determining the exact onset of growth impairments further complicates FGR’s diagnosis and management, as growth may cease at points still deemed within normal ranges. Pathologists and molecular biologists play a pivotal role in such scenarios, examining placental tissues and other samples to uncover pathological factors contributing to growth restrictions, aiding in distinguishing between constitutional smallness and pathological growth restriction [10]. Complicating the definition and management of FGR is the inherent variability in fetal growth patterns, influenced by factors such as ethnicity and parental height [11]. To address this, the medical community is increasingly advocating for personalized growth metrics, or “growth charts”, considering diverse backgrounds and genetic predispositions. Initiatives like the Delphi consensus and the INTERGROWTH-21st project are refining FGR diagnostic and management strategies (Table 1). The Delphi method, with its systematic process, leverages the expertise of a diverse panel to establish consensus on the criteria for diagnosing and managing FGR. The culmination of the Delphi procedure is a consensus-based definition of FGR as a condition in which the fetus fails to achieve its biological growth. This consensus has led to the delineation of specific criteria for identifying early and late FGR. Early FGR, detected before 32 weeks of gestation, is identified through specific measures such as an abdominal circumference (AC) or estimated fetal weight (EFW) falling below the third centile, among other parameters. Late FGR, recognized from 32 weeks’ gestation onwards, is diagnosed through comparable criteria. Apart from growth criteria, the Delphi consensus emphasizes the importance of adequate Doppler blood flows of the umbilical artery, middle cerebral artery and uterine arteries. In tandem with the Delphi consensus, the INTERGROWTH-21st project endeavors to set international standards for fetal growth.

The INTERGROWTH-21st project’s growth charts promise a revolutionary approach to assessing fetal growth, adopting a holistic and inclusive perspective. By incorporating data from varied global populations and considering an array of factors influencing fetal growth, these standards aim to represent the pinnacle of fetal development universally [12]. Additionally, the Hadlock growth charts are an essential tool in prenatal care for evaluating fetal growth [13]. Developed from US measurements, these charts provide healthcare professionals with the means to compare an individual fetus’s growth to established norms, thereby facilitating the diagnosis of FGR. The WHO growth charts proposed a universal approach to assessing fetal growth. These charts were created based on longitudinal data of low-risk pregnancies from 10 countries with different economic statuses. The concept created a framework of fetal growth assessment that was supposed to be adequate for any population. The principal limitation involved the misidentification of fetuses as either growth-restricted or overgrown within specific populations, potentially influencing how clinical management approaches are implemented [14]. A similar limitation was observed regarding NICHD growth charts focusing on the American cohort. Applying these charts outside the United States may require careful consideration [15]. Despite differentiated approaches, the proper diagnosis of hypotrophy, especially late-onset FGR, remains challenging [16].

## 3. Growth Velocity—A New Concept of Abnormal Fetal Growth Diagnosis

The utilization of ultrasonography has undergone profound advancement, particularly in the realm of FGR diagnosis. Nevertheless, the outnumbering of postnatally detected hypotrophy questioned the accuracy and sensitivity of adapted criteria [17]. A novel approach to fetal surveillance proposed by obstetrics was fetal growth velocity. This parameter involved changes in fetal weight or a specific biometric index (e.g., AC or HC) from at least two measurements over an interval. The assessment of its growth deviation from a normal trajectory holds potential clinical significance in distinguishing between constitutional and pathological growth abnormalities in contrast to singular measurements of EFW. More prospective research is needed as there is a shortage of empirical evidence of advances in fetal growth velocity application [18,19].

The origin of the growth velocity concept dates to the 1990s. Guihard-Costa and Larroche observed non-uniform fetal growth patterns across the trimesters. Growth velocities of biparietal diameter (BPD), abdominal transverse diameter (ATD) and femur length (FL) peaked at about 16 weeks, yet afterward, FL displayed a decreasing trend. The body weight exhibited a progressive growth trajectory until approximately 34–35 weeks, followed by a deceleration in the growth rate. A similar pattern was observed in the growth of the brain with the exclusion of the infratentorial region of the brain. That region continued to undergo growth throughout fetal development without experiencing a decline at 35 weeks. Additionally, the hemispheres presented a decreasing velocity trend during the pregnancy [20,21,22]. In 1992, Gardosi’s work introduced the attempt to estimate optimal growth velocity using two consecutive third-trimester scans; however, a primary concern arose from potential measurement errors that may overestimate or underestimate growth [23]. In the following years, the researcher, in cooperation with Mongrel and Mul, reviewed potential alternative methods for determining abnormal fetal growth velocity. The authors presented the concept of calculating the optimal weight that the fetus could have reached by assuming no interference from pathological factors. The assumption was based on physiological coefficients such as gestational age, fetal gender, parity, maternal booking weight, maternal height and ethnic group. Subsequently, the anticipated birthweight was modified either upward or downward from a fixed reference value based on a linear additive model. Finally, the specific curve was delineated, indicating the trajectory of potential growth. A typical pregnancy exhibits substantial resemblances across diverse populations. Consequently, by around 31 weeks, approximately half of the anticipated term weight is attained. By the 28th week, approximately 34% of the term weight is acquired, while by the 37th week, roughly 84% is gained [17,18].

The methods mentioned and novel standards are currently used in research on advances in growth velocity applications. Mongelli et al. introduced a technique for evaluating growth velocity based on the mean weekly increase in fetal weight, assuming that fetal growth in the third trimester is quasi-linear. The average fetal weight gain (AWG) was calculated using the difference between birthweight (BW) and EFW from the US scan in the 24th week divided by the difference between the number of weeks at birth and 24 weeks. Normally distributed AWG was estimated to be 176.5 g/week. The most considerable factors affecting AWG included fetal gender and smoking [21]. Different reasoning regarding improper growth velocity in the AGA group was identified using the Individual Growth Assessment (IGA). This approach relies on estimating the growth potential of individual fetuses using second-trimester growth rates. Moreover, the model does not assume that fetal growth is linear, which makes the approach more accurate. These parameters are further utilized to define Rossavik size models, which generate expected growth trajectories specific to anatomical parameters during the third trimester for each fetus (referred to as individualized standards). By comparing actual measurements to these standards, known as percent deviations (%Dev), growth assessments can be made for each individual at different time points. Evaluating the percentage of deviation outside the normal range (pathological %Dev [%Devp]) provides a measure of growth pathology [7]. The beneficial aspect of the implementation of growth velocity may be used in a more precise diagnosis of intrauterine growth restrictions. A study by J. Gardosi and O. Hugh presented a comprehensive comparative analysis of five fetal growth velocity models, aiming to define slow growth in fetuses and evaluate their effectiveness in identifying stillbirth risk alongside the risk associated with small-for-gestational-age (SGA) fetuses. The models included a fixed velocity limit of 20 g per day (FVL20), a fixed >50 centile drop (FCD50), a fixed >30 centile drop (FCD30), growth trajectory slower than the third customized growth centile limit (GCL3) and EFW at second US scan below the projected optimal weight range (POWR). The study revealed significant variation in how slow growth was defined across the different models, with rates ranging from 0.7% to 19.8%. This highlights the need for more standardization in defining slow growth, necessitating careful consideration of the model used. Among the models evaluated, only the measurement-interval-specific POWR model showed the ability to identify non-SGA fetuses with slow growth at increased risk of stillbirth. This underscored the importance of utilizing models that accurately predict adverse outcomes beyond SGA status. Overall, the study underscores the importance of selecting appropriate models for defining fetal growth velocity and highlights the need for more accurate and effective methods to identify fetuses at risk of APOs. The POWR model stands out as a promising approach, offering a more precise assessment of fetal growth and associated stillbirth risk. These findings advance our understanding of fetal growth dynamics and enhance antenatal care practices to optimize pregnancy outcomes [22]. Secondly, the authors developed a model to project EFW from the first to the second consecutive US measurements along its centile rank. This approach allowed for the definition of normal growth velocity specific to the measurement interval based on partial receiver-operating-characteristics-curve (pROC) analyses for SGA and LGA birthweight. The study demonstrated significant associations between growth velocity and predefined perinatal outcome measures, including stillbirth, neonatal death, SGA, LGA at birth, 5-min Apgar score < 7 and neonatal intensive care unit admission. Slow growth between consecutive scans was particularly notable, being significantly associated with stillbirth and neonatal death. Slow growth between the last two scans, in both SGA and non-SGA at the last scan, was identified as a predictor of stillbirth. It highlights the importance of considering growth velocity in addition to fetal size for accurate risk assessment [23]. Another valuable insight into fetal growth velocity standards involves identifying peak velocity for various fetal biometric parameters, such as head circumference (HC), abdominal circumference (AC) and FL, occurring around 16–17 weeks of gestation. The researchers observed distinct growth patterns for different fetal structures, with rapid velocity reduction towards term, particularly pronounced for FL. These results corresponded to the data presented in the historical work of Guihard-Costa and Larroche. The fractional polynomial regression model was used to study and highlight differential growth patterns between the fetal skeleton and abdomen. While the velocity of AC remained relatively steady throughout pregnancy, other parameters exhibited more marked reductions in velocity towards term. This differentiation underscored the importance of considering distinct growth trajectories for various fetal structures [24].

## 4. Undiagnosed Growth Pathologies among Appropriate-for-Gestational Age Fetuses

Applying the 10th centile as an arbitrary threshold neglects the acknowledgment of appropriate-for-gestational-age (AGA) fetuses and neonates who pose the characteristics of late-growth restriction. In their work, Kennedy et al. underline that 70% of term stillbirths occur in fetuses categorized as AGA. These cases involved fetuses whose weight exceeded the 10th centile, but their growth potential was not met due to placental insufficiency [25]. Such fetuses carry characteristics that correspond to the clinical picture of late-onset FGR, as the etiology of placental complications is particularly enhanced during the last trimester of the pregnancy. The gradual development of the placenta involves the increase in villous capillaries, the appearance of syncytiocapillary membranes and the formation of vasculosyncytial membranes. This proximity shortens the distance between the fetal and maternal blood supplies and sustains fetal well-being in the final period of the pregnancy. Improper development of vascular villous tree and placental membranes may diminish the supply of oxygen and nutritious substances to the fetus, eventually leading to hypoxia [9]. Analogous reasoning regarding improper growth velocity in the AGA group was identified using the Individual Growth Assessment (IGA) previously discussed. Although Deter et al.’s work showed that the percentage of growth restriction was significantly lower in AGA groups than in SGA groups, it was recognized in one-third of the cases [26]. These studies underline the risk of underestimating the rate of inadequate growth despite qualification to the AGA group according to commonly used criteria. An antenatal slow growth trajectory affects postnatal catch-up growth of newborns, which has been linked to potential long-term health issues for newborns. This catch-up was more commonly observed in infants with AGA who experienced slowed growth during pregnancy than those who maintained steady growth in the third trimester. It serves as additional evidence of FGR occurring among fetuses with reduced prenatal growth velocity, even if they are born with appropriate weight for gestational age. This suggests that these infants may constitute an unrecognized group at risk not only for the prenatal, intrapartum and neonatal consequences of FGR but also for health issues in infancy and adulthood associated with FGR [17,27].

An essential subject related to fetal weight is accelerated growth velocity in the AGA group. According to novel studies, the mentioned factor was related to the appearance of shoulder dystocia, a complication typically associated with macrosomia or LGA. In the work of MacDonald et al., accelerated growth velocity was calculated using two US scans within the third trimester (28–36 weeks) and assessed as an increase in weight >30 centiles over eight weeks. Due to the presented results, EFW or AC velocities exhibited greater sensitivity and positive predictive value in predicting shoulder dystocia compared to a single measurement of EFW above the 95th centile at 36 weeks. With each one-centile increase in EFW between 28 and 36 weeks, there was an 8% rise in the odds of shoulder dystocia. Similarly, for every centile increase in AC between 28 and 36 weeks, the odds of shoulder dystocia increased by 9% [28]. As 60% of cases are still not anticipated, this underscores that numerous dystocia cases occur in not-macrosomic infants. Accelerated growth velocity, indicating abnormal excessive growth, could serve as a valuable supplement to binary thresholds of suspected macrosomia in identifying at-risk pregnancies [29]. The research of Simic et al. analyzed accelerated fetal growth in early pregnancy to identify the risk of severe LGA and fetal macrosomia at term. Based on measurements performed in the first and early second trimesters in US scans, the researchers estimated that in mothers with fetuses that were at least seven days larger than expected as compared with mothers without an age discrepancy at the second-trimester scan, the odds of having a severe LGA or macrosomic infant were significantly elevated [30].

## 5. Abnormal Fetal Growth Velocity—A Revolutionary Parameter in the Diagnostic Process

The introduction of growth velocity assessment holds the potential to mitigate aberrant perinatal outcomes and augment diagnostic precision about fetal growth abnormalities encompassing cohorts of both small and large fetuses. The implementation of growth velocity may influence the assessment of the final EFW and the prediction of possible complications during pregnancy and labor. In the study of Rodriguez-Sibaja et al., the abnormal AC growth velocity (ACGV) was a parameter correlated with APOs. This relationship was also observed in a group of newborns with EFW in between 10 and 80 centiles, underlying the accuracy of the growth velocity rather than singular US measurements [31]. The concept of ACGV analysis was also evaluated regarding placental insufficiency within the cohort of AGA fetuses. Kennedy et al. noted that the likelihood of having an umbilical artery pH < 7.15, a small placenta (<10 centile) and a deficiency in body fat percentage escalated with each centile decrease in the ACGV. Also, declines in the ACGV of more than 30 centiles between 20 and 36 weeks were linked to a two- to threefold rise in relative risks concerning these markers of placental insufficiency [25].

Moreover, the relevance of growth velocity in predicting APOs was underlined, as in combination with EFW and CPR at admission, it showed a stronger correlation with APOs compared to any of those individual parameters [6]. Pedersen et al. revealed the association of perinatal mortality with conditional BPD decrease in the second trimester—this relationship was not presented in single US scans [32]. Pacora’s study supported that growth velocity doubled the sensitivity for predicting antepartum fetal death. The importance of parameter application was underlined, as 74% of antepartum fetal deaths were considered AGA fetuses [33]. Regarding the time of delivery, decreased growth velocity among AGA fetuses exhibited a relationship with nonreassuring fetal heart rate (NRFHR) at birth and an increased rate of unplanned Cesarean sections [34].

As previously mentioned in the review, the parameter effectively predicted potential labor complications such as shoulder dystocia. Accelerated growth velocity demonstrated higher sensitivity and positive predictive value in anticipating shoulder dystocia compared to a solitary EFW measurement above 95 centiles at 36 weeks. Each one-centile increase in EFW was associated with an 8% elevation in the odds of shoulder dystocia [30]. Despite the undoubted potential of fetal growth velocity assessment, the researchers indicated the potential limitations of the parameters, presented in Table 2 and Table 3.

A compelling study examining the association between fetal cranial growth trajectories and growth and neurodevelopment outcomes at two years of age presented an interesting aspect of the influence of growth velocity on future consequences. This research, conducted as part of the INTERBIO-21st Fetal Study, sheds light on the critical role of fetal growth in shaping long-term neurodevelopmental outcomes. The study identified five distinct trajectories of fetal cranial growth, each associated with specific neurodevelopmental, behavioral and visual outcomes at two years of age. These associations remained significant even after adjusting for factors such as fetal abdominal growth, postnatal morbidity and anthropometric measures at birth and age 2. Trajectories changed within a 20–25-week GA window, suggesting a critical period during which fetal cranial growth patterns influence neurodevelopmental outcomes [35].

**Table 2:** Advancing fetal health: rethinking growth velocity and percentile chart limitations in perinatology.

Issue	What is Measured	Main Assumptions	References
Fetal growth velocity standards	- Peak velocity observed at 16–17 weeks for HC and AC. - Rapid slowdown in growth velocity for HC, BPD, FOD and FL until due day. - AC velocity—steady throughout pregnancy. - Based on a diverse, low-risk pregnancy cohort.	Establishing international fetal growth velocity standards allows for a more nuanced understanding of fetal development, enhancing global monitoring of fetal health.	(Ohuma et al., 2020) [24]
Impact of PM2.5 exposure on fetal growth velocity	- PM2.5 exposure negatively associated with the velocity of HC, BPD, AC and FL. - Significant velocity decrease observed between 22 and 32 gestational weeks. - Late second trimester to early third trimester identified as potential sensitive exposure window. - Highlights environmental impact on fetal growth.	Prenatal exposure to PM2.5 is detrimental to fetal growth velocity, especially during critical development windows, emphasizing the need for environmental health policies and calls for public health interventions to mitigate exposure to pollutants.	(Cao et al., 2021) [36]
Diagnostic accuracy of growth charts	- A comparison of SGA diagnosis using four common fetal growth charts revealed a significant impact on the detection rate for FGR. - Hadlock chart closest to the expected rate of 10% for SGA diagnosis. - Charts vary significantly in detection and false positive rates for placental pathology associated with FGR.	The selection of fetal growth charts significantly affects the diagnosis of FGR, underscoring the importance of accurate, population-specific references.	(Melamed et al., 2021) [37]
Fetal growth velocity and birth outcomes	- Accelerated third-trimester growth velocity increases the risk of shoulder dystocia among non-LGA fetuses. - Increased odds of shoulder dystocia with each centile increases in EFW and AC between 28 and 36 weeks. - Predicts shoulder dystocia risk better than 36-week EFW >95th centile.	Accelerated fetal growth velocities in the third trimester are linked to increased shoulder dystocia risks, advocating for more vigilant prenatal monitoring and tailored delivery planning.	(MacDonald et al., 2021) [5]
Growth velocity and placental insufficiency	- Reduced growth velocity associated with placental insufficiency in term AGA infants. - Indicators of placental insufficiency linked with declining 20–36-week fetal growth velocity. - Reduced growth velocity associated with cerebral redistribution, neonatal acidosis and low neonatal body fat percentage.	Reduced antenatal growth velocity in AGA fetuses is a significant indicator of placental insufficiency, highlighting the need for enhanced monitoring strategies, such as a routine 36-week ultrasound.	(Kennedy et al., 2020) [25]

Abbreviations: HC—head circumference, AC—abdominal circumference, BPD—biparietal diameter, FOD—occipitofrontal diameter, FL—femur length, FGR—fetal growth restriction, LGA—large for gestational age, EFW—estimated fetal weight, AGA—appropriate for gestational age.

**Table 3:** Key insights on fetal growth velocity and ultrasound biometry.

	Key Points	Key Argument
Fetal growth velocity standards	1. Peak velocity for head circumference and femur length around 16 weeks. 2. Steady growth velocity for abdominal circumference. 3. Rapid slowdown in velocity towards term for most biometrics. 4. Variability in growth velocity among different populations, notably Chinese. 5. Potential utility in predicting adverse perinatal outcomes.	Provides a basis for evaluating fetal health and development.
Impact of environmental factors	1. Exposure to particulate matter reduces fetal growth velocity. 2. Critical exposure window identified between 22 and 32 weeks. 3. Decrease in biometric measurement velocity linked to PM2.5. 4. Sensitive period for fetal development affected by external pollution. 5. Potential for tailored prenatal interventions.	Highlights the environmental impacts on fetal development.
Fetal growth predictors of neonatal outcomes	1. Reduced velocity associated with antepartum fetal death. 2. Discrepancies in growth velocity predictive of SGA neonates. 3. Velocity analysis improves SGA prediction over single measurements. 4. Consistent reduced velocity linked to placental insufficiency. 5. Early detection through velocity monitoring can guide clinical decisions.	Suggests monitoring growth velocity for early intervention.
Advancements in ultrasound technology	1. BiometryNet offers automated, reliable fetal biometry. 2. Dynamic Orientation Determination enhances measurement accuracy. 3. Reduction in operator dependency for ultrasound measurements. 4. Potential for widespread clinical adoption due to robust validation. 5. Supports non-expert use in varied clinical settings.	Improves the accuracy and efficiency of fetal biometry.
Challenges in ultrasound measurement	1. Variability due to sonographer skill level. 2. Difficulty in consistent landmark detection. 3. Challenges in measuring rapidly growing fetal structures. 4. Need for improved training and standardization. 5. Development of automated tools to reduce human error.	Stresses the need for standardized and improved ultrasound training.

The assessment of growth velocity is susceptible, like other methods of measurement used so far, to a potential source of error. One is the skill and experience of the person performing the test, which can affect the accuracy of the measurement. Another aspect to consider is the gestational age at which the measurements are taken. Improvement could be achieved by repeating the measurement, calibrating the equipment and following the universal guidelines regarding EFW assessment. Measuring too early or too late relative to the standard periods assumed in the model can induce an error in determining the growth trajectory.

Moreover, if growth standards are set for one population, they may inappropriately reflect the growth patterns in other ethnic groups. Obstetricians have already noticed this problem when using growth charts. As mentioned, the given population should use adequate and customized growth charts. The potential norm ranges should be adapted to a given population or ethnicity if growth velocity is introduced.

## 6. Conclusions

The detection of fetal growth abnormalities and the prediction of possible perinatal outcomes are ongoing challenges in obstetrical practice. Recent studies have revealed a significant relationship between abnormal growth velocity and adverse perinatal outcomes. By combining growth velocity with other parameters like estimated fetal weight (EFW) and cerebroplacental ratio (CPR), we can enhance the prediction of adverse perinatal outcomes. Furthermore, growth velocity has been associated with perinatal mortality and complications, demonstrating its effectiveness in predicting labor complications. The concept of fetal growth velocity, tracking a change in fetal size over a period, holds immense promise as a parameter for assessing fetal surveillance. This potential should inspire further research and analysis in this area.

## Figures and Tables

**Table 1:** Comparative overview of fetal growth charts: limitations and implications for clinical practice.

Growth Chart	What is Measured	Limitations	Usage
Hadlock (by Hadlock et al.)	EFW	- Do not incorporate specific population differences leading to significant discrepancies in fetal growth assessment across diverse populations. - The accuracy of the Hadlock charts may decrease as pregnancy advances, particularly in the third trimester, leading to challenges in diagnosing conditions like FGR or macrosomia/LGA.	Employed extensively for monitoring fetal development and identifying SGA fetuses. Utilized globally in numerous healthcare settings for prenatal care, offering a standardized approach to estimating fetal weight and assessing growth patterns over time.
INTERGROWTH-21st (by Villar et al.)	Fetal growth in an international population	- Provide international standards for fetal growth based on a diverse population study. - Limited in specific ethnic and geographic groups (eventually reduces effectiveness in accurately reflecting the unique growth patterns). - Possible misclassification of FGR, SGA. - May not suit all local or regional populations, necessitating adjustments, considerations or even the development of supplementary guidelines.	Recommended for application across international and multi-ethnic populations, aiding in the global standardization of fetal growth assessment. Commonly used for international research comparisons and in settings with diverse populations, providing a framework for evaluating fetal growth that transcends regional and ethnic differences.
WHO	Global standards for fetal growth	- Provide a universal standard for assessing fetal growth across the world. - May not account for specific regional, ethnic or environmental factors. - Possible misclassification of FGR, SGA. - May not suit all local or regional populations, yet a necessity for local adaptation may limit the practical utility of the WHO charts in some settings, where fetal growth patterns may differ markedly from the global norms established by the WHO.	Utilized for the standard assessment of fetal growth on a worldwide scale, supporting uniform care practices in prenatal care globally. Supports international health initiatives and research in prenatal and perinatal care, providing a consistent framework for evaluating and comparing fetal growth across diverse international settings.
NICHD	Fetal growth in the American population	- Data based on the American population. - Restricted applicability and relevance of growth charts in non-American settings.	Applied in research and clinical practices within the United States for evaluating fetal growth and identifying potential growth concerns. Used to guide clinical decisions and interventions in pregnancies considered at risk for SGA outcomes within the American healthcare context.
Customized Growth Charts (by Gardosi et al.)	Individual fetal growth considering factors such as ethnicity, maternal weight, height and parity	- Require detailed and accurate maternal and fetal data for customization, including ethnicity, maternal pre-pregnancy weight, height and parity. - Demand for comprehensive data collection. - Necessitates access to specialized software and training for healthcare providers, potentially limiting their widespread use, particularly in resource-constrained environments where such resources may not be readily available.	Enables a personalized approach to monitoring fetal growth, considering individual maternal characteristics and physiological factors. Recommended for tailored fetal growth assessment and management, especially in cases where standard growth charts may not provide accurate reflections of fetal well-being.

Abbreviations: HC—head circumference, AC—abdominal circumference, BPD—biparietal diameter, FOD—occipitofrontal diameter, FL—femur length, FGR—fetal growth restriction, LGA—large for gestational age, SGA—small for gestational age, AGA—appropriate for gestational age, EFW—estimated fetal weight, US—ultrasound, WHO—World Health Organization, NICHD—National Institute of Child Health and Human Development.

## Data Availability

No new data were created.
